# Beneficial Properties of Bromelain

**DOI:** 10.3390/nu13124313

**Published:** 2021-11-29

**Authors:** Pawel Hikisz, Joanna Bernasinska-Slomczewska

**Affiliations:** Department of Molecular Biophysics, Faculty of Biology and Environmental Protection, University of Lodz, ul. Pomorska 141/143, 90-236 Lodz, Poland; joanna.bernasinska@biol.uni.lodz.pl

**Keywords:** bromelain, cancer, inflammation, cardiovascular diseases, coagulation, fibrinolysis, SARS-CoV-2 virus, COVID-19 disease

## Abstract

Bromelain is a major sulfhydryl proteolytic enzyme found in pineapple plants, having multiple activities in many areas of medicine. Due to its low toxicity, high efficiency, high availability, and relative simplicity of acquisition, it is the object of inexhaustible interest of scientists. This review summarizes scientific reports concerning the possible application of bromelain in treating cardiovascular diseases, blood coagulation and fibrinolysis disorders, infectious diseases, inflammation-associated diseases, and many types of cancer. However, for the proper application of such multi-action activities of bromelain, further exploration of the mechanism of its action is needed. It is supposed that the anti-viral, anti-inflammatory, cardioprotective and anti-coagulatory activity of bromelain may become a complementary therapy for COVID-19 and post-COVID-19 patients. During the irrepressible spread of novel variants of the SARS-CoV-2 virus, such beneficial properties of this biomolecule might help prevent escalation and the progression of the COVID-19 disease.

## 1. Introduction

Despite the significant development of modern medicine in many fields such as microbiology, chemotherapy, or cardiology, we are still struggling with the serious problem of the selectivity of the used compounds. The major problem is the high toxicity of the novel pharmaceuticals against normal cells. Thus, one of the most fundamental goals of modern pharmacology is searching for new biologically active compounds that can operate on a wide range of actions with minimal side effects. Recently, a significant increase in interest in plants and the possibility of using natural compounds of plant origin in medicine has been observed. It has been observed that plants are a vital component of our daily diet and an essential source of compounds of natural origin with excellent biological properties. Various medicinal products of plant origin are the foundation of today’s pharmaceutical industry [1]. Due to their high efficiency in many areas of medicine with low side effects, high availability, and relative simplicity of acquisition, we are witnesses to a global revival in the field of interest and use of plant and botanical therapy in health care products [2].

The development of physicochemical methods and research on individual chemical substances isolated from plants has contributed to the vast development of the pharmaceutical sector. It is worth emphasizing that some plant isolated compounds have such significant bio/pharmacological properties that they now function as important drugs. An excellent example of compounds of natural origin commonly used in medicine is analgesic morphine or the chemotherapeutic paclitaxel [2].

A promising compound of plant origin used in many branches of medicine is bromelain, a major sulfhydryl proteolytic enzyme found in pineapple plants (*Ananas comosus*). *Ananas comosus* is one of the most popular, edible tropical fruits, and a member of the family Bromeliaceae, grown in several tropical and subtropical countries, including Thailand, Indonesia, Malaysia, India, Kenya, China, and the Philippines. Due to its distinctive taste, this fruit is an integral part of the diet of many people worldwide. Primarily, for many years, the pineapple was valued because of its pleasant, sweet taste, in addition to a wealth of nutrients such as fiber, numerous vitamins, manganese, and copper. Due to its low calorific value, and at the same time, an enormous wealth of nutrients, it has become a frequent component of diets in people who are concerned with their weight. However, it is worth noting that pineapple and its compounds were formerly successfully used in folk medicine for various health problems [3].

Only about 25% of pineapple can be used as a marketable product in the food industry, whereas 75% (leaves, crown, stem, and bark) is treated as agricultural waste [4]. Plant elements such as leaves, stems, and skin are usually used as animal feed. However, numerous phytochemical studies carried out on leaf extracts of pineapple stem have shown that they also contain many biologically attractive alkaloids, flavonoids, saponins, and tannins. Currently, it is believed that the attractive healing properties of this plant can be attributed to the action of bromelain present in the pineapple. This chemical component of pineapple has the most extensive and complex spectrum of biological activity of all known and isolated chemical substances of this plant. Research has shown that this enzyme is characterized by anti-inflammatory, cardioprotective, immunomodulatory, antioxidant, and anticancer properties. In addition to the clinical approach, bromelain also has applications in many branches of the food industry, such as food industries, breweries, flesh processing, textile, and cosmetics industries. Such a large number of potential applications of bromelain indicates therapeutic value resulting from its biochemical and pharmacological properties, which have generated a substantial increase in interest in this compound among scientists and the pharmaceutical and food industries [3,5]. In recent years, commercial production of bromelain extracts has increased significantly, which has contributed to the development of faster and more effective large-scale enzyme production and purification techniques. Currently, dietary supplements containing bromelain are used in many health currents [6,7].

Despite numerous studies on the biological properties of bromelain, the molecular mechanisms of activity of this enzyme are still not fully understood. Due to the promising results about the use of bromelain in the treatment of cancer, today’s attention is focused on a better understanding of the anticancer properties of this natural substance.

The purpose of this review is to thoroughly discuss the complex biological effects of bromelain and its impact on human health based on the available and latest literature and possible commercial uses of the enzyme in the context of the medical/food industry, in addition to prospects for the future. We hope that this work will offer other researchers relevant and valuable data on bromelain research.

## 2. Methods

This review was based on literature collected by searching the PubMed database of the National Library of Medicine using the following keywords: “bromelain” or “bromelain” and “cancer”, “inflammation”, “cardiovascular diseases”, “coagulation”, “fibrinolysis”, “medicine”, “SARS-CoV-2 virus”. Each of the chosen articles was published prior to 1 October 2021, and written in English. The relevance of the articles was estimated by analyzing their title and abstract. The articles meeting all search criteria were full-text evaluated and included in this review.

## 3. Biochemical Characterization

Proteases (proteinases, peptidases, or proteolytic enzymes) are a complex group of enzymes that have been found in a wide variety of organisms, including plants, animals, and microorganisms. They perform many essential functions in various processes, e.g., digestion, proliferation control, cell growth and death, regulation of protein synthesis, and degradation. They also play an important role in the replication and spread of viruses, bacteria, and parasites. Proteolytic enzymes belong to the class of hydrolases (EC 3.4.). They hydrolyze the peptide bond in the middle of the amino acid chain (endopeptidases) or at its ends (exopeptidases). It is assumed that these enzymes can be divided into three main categories [8]. The first division is due to the substrate and the location of the bond, which is hydrolyzed. The second concerns the mechanism of catalysis. In the third category, the proteases are divided according to the pH at which they show their maximum activity. To date, in the human body, over 500 compounds of this type have been found that are encoded by approximately 2% of all genes [9,10,11].

In modern medicine and biotechnology, plant proteases have been received considerably increased attention because of their exploitable properties. One of the most often recognized plant proteases with superior marketable values is bromelain from pineapple [4,6].

Bromelain, the primary protease of *Ananas comosus*, is referred to as a crude aqueous extract obtained commercially from the stem of pineapple fruit. It has been known chemically since 1876 and the work on isolation of bromelain began in 1894 [12,13]. In biochemical terms, bromelain is a non-toxic compound with therapeutic values, classified as a protein-digesting enzyme protease. It is worth noting that the bromelain extract, in addition to various thiol endopeptidases, also includes other components such as phosphatases, glucosidase, cellulases, peroxidases, glycoproteins, carbohydrates, several protease inhibitors, and organically bound Ca^2+^ [14,15]. It is assumed that the percentage composition of the bromelain extract consists of 80% stem bromelain, 10% fruit bromelain, 5% ananain, and other ingredients [16].

Depending on the part of the fruit from which the enzyme was isolated, two types of enzymes are distinguished—stem bromelain (SBM), which is assigned the EC number EC 3.4.22.32, and fruit bromelain (FBM) with its EC number EC 3.4.22.33. Slightly unfortunate names may suggest that they represent two forms of one enzyme. However, research has shown that SBM and FBM differ in biological activity, structure, and physicochemical properties [4,12,17]. Practically only SBM is used in the pharmaceutical, medical, and food industries. Acquiring bromelain from this part of the pineapple is much more profitable because enzyme concentration in the pineapple stems is higher than in the fruit part. Moreover, the complex process of extraction and purification of SBM is cheaper [6,18]

The molecular weight range for SBM is 26–37 kDa and for FBM the molecular weight range is 24.5–32 kDa [7,18,19,20]. Significant data on biochemical analysis of the SBM extract is provided by Herrach et al. 1995 [21]. Application of two-step cation-exchange chromatography allowed isolation of nine basic proteolytically active SBM extract components. F4 (24.4 kDa) and F5 (24.5 kDa) are two main components estimated to be 50% glycosylated. They are stabilized by disulfide bridges and numerous hydrogen bonds [22]. The most active protease in the SBM extract appears to be the fraction termed F9 (ananain; 23.5 kDa), representing about 2% of total proteins. Studies have shown that F9 is not glycosylated [21]. Differences in the functioning of SBM and FBM are also manifested in the disproportion of the optimal pH and environmental temperature, factors that affect the activity of pineapple extracts [12]. Studies have shown that for SBM, the appropriate pH is between 6 and 7, and the optimum temperature range is 50–60 °C [23,24,25]. For FBM, the optimum pH range is 3–8, and the best temperature is in the 37–70 °C range [4,26,27].

Due to its wide application in the food, pharmaceutical, biotechnology, and medical industries, bromelain should be characterized by low systemic toxicity and good absorption in the body while maintaining sufficiently high biological activity. Animal experiments have shown that bromelain has very low toxicity with a lethal dose (LD) greater than 10 g/kg body weight. In dogs and rats treated with the extract, its cytotoxic or carcinogenic effects have not been demonstrated [28]. Clinical tests on patients also showed no undesirable side effects of bromelain. However, it is worth noting that it is likely that in people with hypertension, long-term bromelain administration may lead to tachycardia [29]. Our research (unpublished data) also showed that bromelain is not cytotoxic to normal endothelial cells (HUVECs) isolated from the human umbilical vein. Cytotoxicity tests were performed using a resazurin reduction assay. Bromelain did not affect HUVECs survival even at a maximum concentration of 50 µg/mL.

Research suggests that some people may experience allergic reactions when using bromelain. People who are allergic to pineapple are particularly vulnerable [30]. In such people, bromelain can cause IgE-mediated respiratory allergies manifested in difficulty breathing, clogged sinuses, angioedema, wheezing, and coughing [31,32,33]. 

Absorption of bromelain occurs mainly through the digestive tract. It is estimated that approximately 40% of functionally intact bromelain may be present in the blood of rats after oral administration of the extract, with the highest concentration observed up to an hour after administration [34,35]. Castell et al. [34] showed that bromelain has a half-life of 6–9 h. Moreover, bromelain present in the plasma retained its proteolytic properties and was linked to two blood proteins—alpha 1-antichymotrypsin and alpha 2-macroglobulin, which stabilizes even dilute solutions of bromelain. In an artificial stomach juice, the bromelain concentration of 3.66 mg/mL was detected after 4 h and it remained in artificial blood at a concentration of 2.44 mg/mL after 4 h [36].

Due to its good biological functions, lack of systemic cytotoxicity, and often excellent benefits that it offers, bromelain has been permanently used in many industries, such as cosmetology, pharmacy, food, and biotechnology. Furthermore, increasing scientific data indicate the interesting antitumor properties of bromelain extracts that support the action of available chemotherapeutic agents. The commercial use of bromelain has contributed to the development of effective methods for extracting the enzyme from the pineapple stem, and the resulting preparations are characterized by ultra-purity. In this review, we focus on the use of bromelain in the medical industry and discuss the most important biological properties of the enzyme concerning disorders of the human body.

## 4. Bromelain in Cardiovascular Diseases

Cardiovascular diseases (CVDs), including coronary heart disease (heart attacks), disorders of the blood vessels, cerebrovascular disease (stroke), raised blood pressure, angina pectoris, blood coagulation, and fibrinolysis disorders, have been a severe global problem for modern medicine for a long time. Modern medicine is constantly looking for new treatment strategies to reduce the costs and cardiovascular complications, and more importantly, to slow the progression of CVDs [37]. High hopes are associated with natural compounds of plant origin, usually characterized by effective biological effects with very low systemic side effects, which are often smaller than those of synthetic drugs [38,39]. Systematic studies suggest that bromelain, due to its biological properties, is an attractive compound in treating several CVDs [23,40,41].

### 4.1. Ischemia/Reperfusion

One of the most commonly diagnosed cardiovascular diseases is coronary artery disease (myocardial ischemia-reperfusion I/R), which develops due to myocardial hypoxia. The main reasons for the development of coronary heart disease are changes in the coronary arteries that impede blood flow [42]. Despite significant progress in the treatment of heart disease, new and effective treatment strategies are constantly being sought. High hopes are associated with compounds of natural origin, including bromelain [43].

Several in vitro and in vivo studies indicate that bromelain at appropriate doses may reduce or minimize symptoms associated with several cardiovascular diseases [44,45]. Due to its anticoagulant and fibrinolytic properties, bromelain is used for the prevention and treatment of thrombophlebitis. Bromelain induces the disruption of thrombus, reduces the platelets clumping and blood viscosity [46]. Due to its anticoagulant and fibrinolytic properties, bromelain is used for the prevention and treatment of thrombophlebitis. Bromelain induces the disruption of thrombus, reduces the platelets clumping and blood viscosity [46]. It also prevents or minimizes the severity of angina pectoris and transient ischemic attack (TIA) [47]. In vivo studies showed that bromelain, due to its excellent fibrinolytic properties, caused the dissolution of atherosclerotic plaque with high efficiency, thereby reducing the risk of atherosclerotic disease. In addition, 73 patients with acute thrombophlebitis given bromelain in combination with painkillers experienced a significant decrease in inflammatory symptoms such as pain, swelling, and high temperature [48]. 

Extremely relevant information on the molecular mechanisms of bromelain activity in cardioprotection against I/R was provided by the study of Juhasz et al. [49]. In ex vivo studies in an isolated rat heart model, they examined the effectiveness of bromelain treatment on the degree of I/R injury. Myocardial ischemia results in the apoptosis of cardiomyocytes and leads to severe damage. During the disease, Akt kinase and the members of the forkhead box transcription factor/protein (FOXO) family (FOXO1, FOXO3a, and FOXO4) are involved in regulating the programmed cardiomyocyte death through the Akt-FOXO signaling cascade [50,51].

Studies have shown [49] that bromelain treatment resulted in a statistically significant improvement in left ventricular function during reperfusion. Furthermore, it was found that aortic flow was increased compared to untreated rats. Bromelain-mediated cardioprotection results from programmed cell death (PCD) inhibition after its administration, probably by its activation effects on the Akt-FOXO signaling pathway. Research indicates that bromelain caused an increase in phosphorylation of Akt kinase (active form) and its translocation to the cell nucleus, which leads to the phosphorylation of FOXO3A, thereby cell death signal is inhibited.

It is worth emphasizing that the protective effect of bromelain during I/R injury was also observed in the ex vivo research in rat liver [52] or rabbit skeletal muscles models [53]. Prevention of lack of flow and reduction in interstitial edema, improved microcirculation, and preservation of muscle tissue were observed

### 4.2. Blood Coagulation and Fibrinolysis

Studies on the anticoagulant properties of bromelain were carried out as early as in the second half of the 19th century, almost 70 years ago [54]. The ex vivo studies were performed on patients after myocardial infarction, stroke, or high aggregation value orally administered bromelain. It has been shown that bromelain causes a decrease in platelet aggregation, thus reducing the risk of arterial thrombosis and embolism. These results were confirmed in later numerous studies conducted both in vitro and in vivo, where bromelain was shown to cause platelet inhibition in a dose-dependent manner. [55,56].

Bromelain acts on both the external and internal pathways of the blood clotting system. Bromelain regulates blood coagulation homeostasis by inhibiting fibrin synthesis and by increasing serum fibrinolytic activity. Relevant data was provided by Errasti et al. [57], who observed that bromelain showed a dual effect on blood coagulation depending on the concentration used—a procoagulant effect at low concentrations, whereas an anticoagulant effect was noted at high concentrations.

At high concentrations of bromelain, inhibition of both prothrombin to thrombin conversion was observed, resulting in prolonged prothrombin time (PT) and activated partial thromboplastin time (APTT), and a decrease in ADP-induced platelet aggregation [55,58]. Moreover, Metzig et al. [56], in studies on platelet aggregation and adhesion to endothelial cells, showed that after incubation of platelets with bromelain before activation with thrombin, aggregation was stopped entirely.

In vitro and in vivo studies have shown that bromelain is also a potent fibrinolytic agent. It stimulates the increased conversion of plasminogen to plasmin, resulting in increased degradation of fibrin [59]. The mechanism of the fibrinolytic and anticoagulant activity of bromelain is still not fully understood, but it is believed that it is associated with its proteolytic activity [46,60]. The latest research indicates that the bromelain’s fibrinolytic activity and anticoagulant activity are also affected by the appropriate physical processes used during extraction and purification. The use of ethanol extraction [61] or high-pressure processing [62] resulted in increased bromelain activity.

Studies show that anti-inflammatory, proteolytic drugs that inhibit platelet aggregation and prostaglandin synthesis are also characterized by good antitumor activity [63,64]. An excellent example of such a drug is aspirin, which, according to recent research, in addition to its antiplatelet activity, is used as a potent chemotherapy agent [65,66,67]. It is believed that fibrinolytic properties and antiplatelet activity may be involved in the antitumor activity of bromelain. Chobotova et al. [60] hypothesize that the inhibitory effect of bromelain on cancer development is due to the prevention of tumor-platelet aggregates. 

Based on several in vitro and in vivo studies, it has also been shown that bromelain can affect blood coagulation by selectively modulating the level of prostaglandin E2 (PGE_2_) and thromboxane A_2_ belonging to prostaglandins. Bromelain, in a dose-dependent manner, caused a statistically significant decrease in the level of PGE_2_ and thromboxane A_2_ activity (their inhibition) and shifted the ratio of thromboxane A_2_/prostacyclin (PGI_2_) in favor of the anti-inflammatory prostacyclin PGI_2_ [23,68,69].

## 5. Antimicrobial Activity

Microorganisms that function in the life of plants and animals in almost every aspect, in addition to their undeniable positive functions, often constitute a source of severe infections and diseases leading to death. In addition, a rapid expansion of microorganisms resistant to commercially used antibiotics is observed. Hence, it is important to develop new treatment methods using natural compounds.

### 5.1. Diarrhea 

Diarrhea is a common gastrointestinal disease that is extremely dangerous for young animals, in addition to new-borns and children. Enetherotoxins released by *Vibrio cholerae* and enterotoxigenic *Escherichia coli* are responsible for diarrhea development [70,71,72]. Studies have shown that in animals supplemented with bromelain, the compound exhibited antibacterial activity. Significant inhibition of bacterial toxin secretion was observed (35–62% decrease depending on the species of the microorganism). Two molecular mechanisms are thought to be responsible for bromelain’s antisecretory properties—inhibition of cAMP and cGMP signaling pathways activated by bacterial toxins [73] and enzymatic modification of the sites of glycoprotein receptor binding with *E. coli* located on the intestinal mucosa, thus preventing bacterial adhesion to intestinal cells [72,74].

### 5.2. Bromelain in Antibiotic Therapy

Due to its good proteolytic properties and very low systemic cytotoxicity, bromelain has found wide application in treatment with antibiotics as a substance that increases their activity. It has been shown that antibiotic therapy combined with bromelain revealed increased effectiveness (in contrast to the antibiotic used alone) in many disease states, e.g., sinusitis, bronchitis, pneumonia, pyelonephritis, thrombophlebitis, perirectal and rectal obsesses, or cutaneous infections caused by *Staphylococcus* [75,76]. In rat experiments, an increase in penicillin and gentamicin concentrations has been observed [77]. Similar results were obtained in humans, where bromelain administration during antibiotic therapy increased the concentration of antibiotics in the blood and urine and resulted in higher blood and tissue levels of tetracycline and amoxicillin [78,79]. Shahid et al. [80] showed that a combination of bromelain, rutin, and trypsin, when used as adjunctive therapy in treating sepsis in children, caused the increased activity of antibiotics. The obtained results indicate that using bromelain, rutin, and trypsin combined with antibiotics is an effective adjunct treatment for early improvement in children and adolescents with sepsis.

In recent in vivo studies, Cai et al. [81] analyzed the effects of the combination of *Serenoa repens*, selenium and lycopene extract + bromelain and methylsulfonylmethane extract associated with levofloxacin in patients with chronic bacterial prostatitis (CBP). The combination of extracts improved the clinical efficacy of levofloxacin in patients affected by CBP. Additionally, an improvement in the quality of life has been observed, which may be related to the anti-inflammatory properties of bromelain. It is noteworthy that during therapy, no side effects were observed.

An innovative approach presenting pharmaceutical nanotechnology was proposed by Khan et al. [82], who offered the synthesis of gold nanoparticles (GNPs) of best qualities in size, stability, and shape using bromelain as a reducing and capping agent. Based on these studies, Bagga et al. [83] developed bromelain-capped gold nanoparticles as drug delivery carriers of levofloxacin. The bromelain-capped gold nanoparticles with levofloxacin and pure levofloxacin were determined by evaluating minimum inhibitory concentration (MIC) against *E. coli* and *S. aureus*. The bioconjugated bromelain-capped GNPs exhibited superior antibacterial activity against both bacteria compared to pure levofloxacin (LvN). The authors suggest that these results indicate superior stability and transport of many LvN molecules into a highly localized area at the site of particle-bacterium contact.

The use of bromelain as a reducing and capping agent in the preparation of nanoformulation of cefotaxime using GNPs was also proposed by Shaik et al. [84]. Antibacterial properties of cefotaxime conjugated gold nanoparticles have been tested on two resistant bacterial strains—*Escherichia coli* and *Klebsiella pneumoniae*. When cefotaxime was used alone, both *E. coli* and *K. pneumoniae* were completely resistant to the antibiotic. The use of cefotaxime-conjugated gold nanoparticles significantly increases the activity of the antibiotic, and inhibition of multiplication of both bacterial strains was observed.

The mechanism of action of bromelain to increase the absorption and activity of antibiotics is not yet fully understood. Grabovac et al. [85] showed that bromelain, in combination with heparin as a stable complex, caused a significant increase in uptake of heparin in Caco-2 cells. The authors suggest that the synergy of action between bromelain and other compounds (including antibiotics) is associated with its proteolytic properties. Bromelain can modify the permeability of organs and tissues for various drugs through changes in the structure of endothelial cells connected by adherents, and tight and gap junctions. As a result of modifying the permeability of organs and tissues for various drugs, bromelain causes an increase in their absorption at the site of infection, thereby enhancing the effectiveness.

### 5.3. Anthelmintic Efficacy

Gastrointestinal infections are a serious global problem for both humans and farm animals. In livestock, they are one of the most significant barriers to animal production, directly affecting performance and leading to high economic losses [22,86,87]. The growing resistance of nematodes to commercially available drugs demand the development of new, alternative treatment methods. Recently, an increase in interest in compounds of plant origin has been observed [88].

In several in vitro and in vivo studies, bromelain has been shown [87,89,90,91] to exert an anthelmintic effect against gastrointestinal nematodes. The obtained results indicate that proteolytic properties are responsible for the antiparasitic activity of bromelain. Bromelain by enzymatic digestion of structural proteins present in the nematode’s cuticle leads to damage and the final loss of motility by parasites. A novel approach to the use of bromelain in the treatment of parasitic nematodes was proposed by Wasso et al. [92], who evaluated in vitro and in vivo the anthelmintic efficacy of chitosan encapsulated bromelain against *Haemonchus contortus*.

Domingues et al. [93] and Luoga et al. [94]. draw attention to the difference in the anthelmintic activity of bromelain, which is weaker in vivo compared to in vitro. Bromelain activity may be affected by the acidic environment of the rumen digestive tract, in addition to the microbial and protozoa fauna present in the intestine. A unique proposal to stabilize bromelain for therapeutic applications is presented in the latest research by Nwagu and Ugwuodo [95] concerning exposure to buffers with a wide pH range and high temperatures.

### 5.4. Antifungal Properties

The microbial activity of bromelain is also manifested in its antifungal properties. Brakebusch et al. [96] indicated that bromelain administered with trypsin was fungicidal against *Candida albicans*. It has been shown that bromelain and trypsin significantly accelerated phagocytosis and respiratory burst killing of *Candida albicans*. 

Lopez-Garcia et al. [97] indicate that bromelain exhibit a potent antifungal property against agronomically important fungal pathogens: *Fusarium verticillioides*, *Fusarium oxysporum*, and *Fusarium proliferatum*, which are the causal agents of diseases in economically important crops. Moreover, *F. verticillioides* is responsible for producing fumonisin B1, which is a very toxic human carcinogen. Because the cysteine protease inhibitor suppresses the activity of bromelain against *F. verticillioides*, *F. oxysporum,* and *F. proliferatum*, the authors point out that its antifungal properties are determined by proteolytic activity.

### 5.5. Antibacterial Properties

Several groups have provided significant evidence for the antibacterial efficacy of bromelain in a variety of classical experiments using different bacterial species [98]. The data provided by Zharfan [99] is fascinating because it gives essential information about the antibacterial properties of pineapple extract (*Ananas comosus* L. *Merr*) containing saponins and bromelain. Using multidrug-resistant *Pseudomonas aeruginosa*, the authors demonstrated that pineapple extract inhibited bacterial growth. Zharfan et al. [99] accept the hypothesis that bromelain, as a proteolytic enzyme, induces enzymatic protein breakdown in the surface membrane, which ultimately weakens the cell wall, leads to cell leakage, swells, breaks down in the bacterial membrane, and damages the cell. However, the authors emphasize that bromelain’s exact mechanism of action on Gram-negative bacteria is not fully understood.

The data on the antibacterial efficacy of bromelain indicate its excellent activity against bacteria, especially common during oral infections—*Enterococcus faecalis* [100,101], *Streptococcus mutans* [101,102,103,104], *Streptococcus sanguis* [102], and *Staphylococcus aureus* [105]—such as periodontitis, infected root canals, periarticular abscesses, or dental caries. In all cases, bromelain has been shown to have a strong antibacterial effect, leading to inhibition of bacterial growth and death. It is worth noting that, as research from Anjos et al. [106,107] shows, bromelain may also find application in the food industry due to its significant antimicrobial potential against strains of *Alicyclobacillus*. Bromelain showed effective inhibitory and bactericidal activity at low concentrations against the species of *Alicyclobacillus*. 

A new approach to the antibacterial use of bromelain was proposed by Ataide et al. [108]. The authors used bacterial nanocellulose loaded with bromelain. Evaluation of the antimicrobial activity of bromelain against *E. coli*, *S. aureus*, and *P. aureoginosa* showed its significant antimicrobial activity. Moreover, incorporation into bacterial nanocellulose potentiates antimicrobial activity.

Ahamed et al. [101] raise the important issue of increasing the antibacterial activity of bromelain extracts depending on the appropriate purification process. Significant results obtained by Amini et al. [109] and Hidayat et al. [110] provide evidence that the appropriate process of physico-chemical purification of bromelain extract affects the effectiveness of the antibacterial activity.

## 6. Immunomodulatory Effect of Bromelain

Inflammation is a biochemically ordered, natural defense mechanism in our body that arises as to the body’s response to an external or internal damaging (pathogenic) factor. The aim of the operation of complex biochemical signaling pathways during inflammatory responses is to defend the organism. The inflammatory process should therefore remove the pathogen and allow the tissue to return to its physiological state. A properly functioning immune system effectively eliminates pathogens without damaging its own cells and tissues. However, immunoregulatory mechanisms can lead to chronic pathological conditions—there is an inflammatory response to its own antigens. This condition occurs in many autoimmune diseases [111]. The need to look for new anti-inflammatory substances is still very urgent due to the unacceptable side effects of commercial anti-inflammatory drugs. High hopes are associated with natural compounds of plant origin, which, as indicated by numerous in vitro studies, very often have good anti-inflammatory activity with very low systemic cytotoxicity. Bromelain seems to be an attractive solution for treating inflammation occurring in different diseases during the neoplastic process [112]. 

Numerous studies indicate that bromelain has very complex immunomodulatory properties realized at many levels of molecular signaling pathways and control of gene expression involved in the immune response. Bromelain has a double effect on modulating the immunological response, which is significant for the proper immune system functioning and preservation of homeostasis. Depending on the microenvironment of the cell, presence of inflammation-inducing conditions, and, finally, the general state of health, bromelain may cause both an increase and a decrease in the activity/expression of the same molecules involved in the immune response [40,60]. Here we present the use of bromelain as an anti-inflammatory factor and its ability to modulate the immune system in diseases and cancers. The most important immunomodulatory activities of bromelain are summarized in Table 1.

### 6.1. Lipopolysaccharide Stimulation

The subjects of numerous studies are bromelain’s immunomodulatory and anti-inflammatory properties and its effect on the production/activity of cytokines involved in the inflammatory process. Using various in vitro research models, both human [113,114] and rat/mouse cells [115,116,117,118], during induction of inflammation by lipopolysaccharide (LPS) stimulation, bromelain has been shown to inhibit the activity of key cytokines and molecules for this process. LPS is an endotoxin from Gram-negative bacteria, which induces inflammation and, hence, the immune response in the form of activation of several intracellular signaling pathways, including the tumor necrosis factor-alpha (TNF-α), nuclear factor kappa-light-chain-enhancer of activated B cells (NF-κB), and several mitogen-activated protein kinase (MAPK) pathways.

Hou et al. [117] and Huang et al. [113] provided important data on the effect of bromelain on the production of cytokines from LPS-stimulated cells, respectively, in rat models and human peripheral blood mononuclear cells (PBMC) or THP-1 monocytic leukemia cells. It has been shown that bromelain in a dose-dependent manner caused significant suppression of interleukin (IL) IL-6, IL-1β, and TNF-α production. In addition, bromelain treatment increased both the inhibition of NF-κB transcription factor and MAPK pathways, resulting in a decrease in cyclooxygenase-2 (COX-2), PGE_2_ mRNA levels, and extracellular signal-regulated kinase (ERK), c-Jun N-terminal kinase (JNK), and p38 activation. The evidence of inhibition of the MAPK kinase pathway is also a bromelain-dependent suppression of activator protein 1 (AP-1) transcription factor activity, which is involved in, among others, apoptosis, cell proliferation, and inflammation. The activity of AP-1, a heterodimer composed of c-Jun and c-Fos, is dependent on the degree of c-Jun phosphorylation and c-Fos expression. Bromelain significantly suppressed the induction of c-Fos and reduced phosphorylation of c-Jun [116]. It is worth emphasizing that using a specific cysteine protease inhibitor resulted in a significant reduction in bromelain suppressive properties relative to the substances mentioned above [113]. A similar anti-inflammatory effect of bromelain has also been observed in rat primary microglial cells [118], human U937 macrophages [114], and RAW 264.7 cells [116]. Moreover, bromelain exhibits anti-inflammatory properties in macrophages by inhibiting the expression of essential pro-inflammatory cytokines and chemokines including macrophage inflammatory protein 1alpha (MIP-1α), macrophage inflammatory protein-1beta (MIP-1β), monocyte chemoattractant protein-1 (MCP-1), IL-8, IL-1β, IL-6, and the cyclooxygenase pathway [117,118].

Referring to the recent studies of Habashi et al. [118] and Lee et al. [116], it is worth emphasizing the inhibitory effect of bromelain treatment on the expression of inducible nitric oxide synthase (iNOS). This enzyme synthesizes nitric oxide (NO), an intercellular signaling molecule involved in several physiological processes regulating homeostasis. By comparison, excessive iNOS activity is observed in cancer [119] and autoimmune diseases [120]. Western blotting and reverse transcriptase-polymerase chain reaction (RT-PCR) analyses for iNOS showed that bromelain inhibited LPS-induced iNOS expression in a dose-dependent manner [116,118].

### 6.2. Chronic Inflammatory Bowel Disease

Due to the good anti-inflammatory properties of bromelain and promising in vitro results, high hopes are associated with its use in treating chronic inflammatory bowel disease (IBD). Numerous studies indicate that bromelain inhibits intestinal inflammation by modulating the expression/activity of compounds involved in inflammation [121,122,123,124,125]. Chronic inflammation of the intestines is a severe disease in patients with ulcerative colitis and Crohn’s disease. Autoimmune bowel disease and the accompanying chronic inflammation can often lead to colorectal cancer [126].

In one of the first in vitro studies on the IBD model, Hale et al. [123] showed that bromelain administered orally to mice caused both a decrease in the incidence and severity of spontaneous colitis and colonic inflammation in piroxicam-exposed IL-10-deficient mice (IL-10^−/−^) with established colitis. Significantly, the effective anti-inflammatory activity of bromelain was associated with a lack of cytotoxicity in mice. The anti-inflammatory effect of bromelain in IBD was confirmed in subsequent in vitro studies by Hale et al. [125]. The authors again showed that long-term dietary supplementation with pineapple decreases colon inflammation and neoplasia in IL-10^‒/‒^mice with chronic colitis. One of the mechanisms by which bromelain inhibits intestinal inflammation may result from its enzymatic effect on the complex process of lymphocyte activation. Flow cytometry studies have shown that bromelain treatment of mouse splenocytes caused proteolytic degradation of several cell surface molecules that are important in immune response due to involvement in leukocyte migration, adhesion, and activation. The differentiation clusters (CD) CD44, CD45R, CD62L, and CD8 were found to be partially sensitive to enzymatic bromelain-dependent degradation.

The anti-inflammatory properties of bromelain in chronic inflammation of the intestine have been confirmed during in vitro [124] studies using human cells from endoscopic colon biopsies from patients with ulcerative colitis and Crohn’s disease. In the course of these diseases, significant activation of the immune system associated with the overexpression of growth factors, metalloproteinases, and pro-inflammatory cytokines was observed. Therefore, the authors examined the secretion of pro-inflammatory cytokines and chemokines after treatment with bromelain, whose overexpression is characteristic of ulcerative colitis and Crohn’s disease. According to the results, bromelain caused a significant decrease in the level of secretion of granulocyte colony-stimulating factor (G-CSF), granulocyte-macrophage colony-stimulating factor (GM-CSF), IFN-γ, CCL4/macrophage inhibitory protein (MIP)-1β, and TNF-α. These cytokines are increased in media derived from organ cultures of colon tissues from patients with active IBD and play a crucial role in the pathogenesis of IBD. Furthermore, the authors suggest that bromelain may reduce leukocyte migration into the colon and thus reduce overall inflammatory activity.

Wen et al. [121] also noted the inhibitory activity of bromelain against iNOS in the colon of postoperative rats. Upregulated colonic iNOS gene expression via the NF-κB pathway has been demonstrated to play an essential role in intestinal propulsive dysmotility of ileus. Treatment with bromelain resulted in significant inhibition of the overexpressed iNOS mRNA in the postoperative rats, which at the same time was associated with the improvement by bromelain of the decreased defecation.

Valuable information on the anti-inflammatory properties of bromelain is provided by the latest in vitro research by Zhou et al. [122]. Scientists using the rat colitis model, intestinal endothelial cell line IEC-6, and human colon epithelial cell line, Caco-2-, investigated the effect of bromelain on the inhibition of transmembrane tumor necrosis factor receptors 1/2 (TNFR1) and (TNFR2) and thus its ability to inhibit the TNF-α signaling pathway. Colitis was determined by administering 2,4,6-trinitrobenzene sulfonic acid, which resulted in tight junction barrier dysfunction, visible macroscopic damage, and a significant increase in NF-κB, TNFR1, and TNRF2 expression levels, which are involved in the inflammatory response in IBD. After treatment with purified bromelain, significant inhibition of TRNF receptors and a decrease in their expression have been observed. In addition, it is worth emphasizing that colitis symptoms, including cytokine profiles, epithelial cell apoptosis, and epithelial tight junction barrier dysfunction, were prominently ameliorated by bromelain. 

### 6.3. Modulation of Leukocyte Adhesion and Activation 

Extremely relevant data on this topic is provided by in vitro studies by Hale et al. [127,128]. The summary of both experiments is a piece of significant evidence that bromelain treatment of leukocytes in whole blood causes a dose-dependent decrease in expression 14 of 59 leukocyte markers studied: CD7, CD8α, CD14, CD16, CD21, CD41, CD42a, CD44, CD45RA, CD48, CD57, CD62L, CD128a, and CD128b. All these bromelain-sensitive cell surface molecules that are moderately to highly expressed on resting peripheral blood lymphocytes, monocytes, or granulocytes play an essential role in leukocyte adhesion, migration, and/or activation during inflammation. Furthermore, studies also confirmed that the activity of bromelain against a decrease in CD antigen expression is due to the enzyme’s proteolytic properties. The use of a proteinase inhibitor—α2-macroglobulin (α2M)—usually present in plasma, caused a significant decrease in bromelain proteolytic reactivity against leukocytes CDs, and no decrease in their bromelain-dependent concentration was observed.

The effects of bromelain on the decrease in expression of cell surface molecules involved in leukocyte activation/migration reported by Hale et al. are in agreement with several subsequent in vivo and in vitro studies [129,130]. Secor et al. [130] demonstrated that bromelain treatment reduces CD25 expression on activated CD4+ T cells in vitro, both in a dose- and time-dependent manner. CD25 is part of the receptor for IL-2 and is considered a crucial therapeutic target in inflammation, autoimmunity, and allergy. As in the case of Hale et al. research [128], bromelain-dependent proteolytic cleavage of CD25 from activated CD4+ T cells and a decrease in CD25 expression was significantly abrogated by the use of a cysteine protease inhibitor. These results indicate the anti-inflammatory potential of bromelain resulting from modulating the activity of one of the most important regulators of immune function, IL-2. Fitzhugh et al. [129] examined the in vitro and in vivo effects of bromelain on the expression of two surface markers CD128a/CXCR1 and CD128b/CXCR2, forming the receptor for IL-8, a chemokine that regulates neutrophil activation/chemotaxis to sites of acute inflammation. Studies demonstrated that during inflammatory stimuli, proteolytic bromelain-dependent removal of the CD128a/CXCR1 and CD128b/CXCR2 receptors reduces chemokine-mediated changes in integrin affinity, resulting in the decreased neutrophils adhesion. Considering the function of surface antigens during the immune response, it is clear that the molecular mechanisms by which bromelain can affect the course of inflammatory diseases may include effects on migration and activation of leukocytes.

### 6.4. Bromelain as an Immunostimulatory Factor

In contrast to the anti-inflammatory properties of bromelain described above, such as inhibition/reduction in expression transcriptional factors, chemokines, cytokines, and receptors crucial for the inflammation process, several studies indicate a bromelain-dependent increase in immune system activity [131,132,133]. The dualism of immunomodulatory activity of bromelain is observed in both in vitro and in vivo studies using PBMC from healthy donors and mice. In one of the first studies, it was shown that in PBMC primed ex vivo with interferon gamma (INF-γ), after treatment with bromelain, TNF-α, the expression level of IL-1β and IL-6 increased [133]. These results were also confirmed in later studies of Barth et al. [132]. Bromelain induced a significant increase in PBMCs of healthy donors of the macrophage/monocyte associated cytokines interleukin IL-6, IL-1β, granulocyte-macrophage-colony stimulating factor (GM-CSF), IFN-γ, and TNF-α. Engwerda et al. [131], in in vitro and in vivo experiments using T and B cells isolated from mouse spleens, showed that bromelain can simultaneously enhance and inhibit T cell responses. The stimulatory effect of bromelain increased T cell proliferation and, in addition, an increase in the T cell costimulatory activity of splenocytes and purified B cells. In conclusion, by activating immune system cells, bromelain can stimulate the innate and adaptive immune systems in response to various disorders.

**Table 1:** Cellular and molecular targets of bromelain related to its anti-inflammatory and immunomodulatory activity.

Target	Experimental Approach	Effect	References
PBMC human peripheral blood mononuclear cells; THP-1 monocytic leukemia cells; U937 human macrophages cells	In vitro bromelain treatment + lipopolysaccharide (LPS)	Anti-inflammatory activity: TNF-α ↓, IL-1β ↓, IL-6 ↓, IL-8 ↓, NF-κB ↓, COX-2 ↓, PGE_2_ ↓, thromboxane B_2_ ↓, macrophage inflammatory protein-1α/β (MIP-1α/β) ↓, monocyte chemoattractant protein-1 (MCP-1) ↓	[113,114]
RAW264.7 mouse monocyte macrophage cells; Primary microglial cells from cerebral cortices	*I*n vitro bromelain treatment + LPS	Anti-inflammatory activity: IL-6 ↓, NF-κB ↓, COX-2 ↓, PGE_2_ ↓, iNOS ↓, Alterations in the expression of MAPK family proteins: p-JNK ↓, p-p38 ↓, p-ERK ½ ↓, c-jun ↓, c-fos ↓	[115,116,117,118,121]
Male Sprague-Dawley (SD) rats; BV-2 mouse microglial cells; Sprague-Dawley male rats	In vivo bromelain treatment + LPS In vitro bromelain treatment + LPS	Anti-inflammatory activity: NF-κB ↓, COX-2 ↓, PGE_2_ ↓ Alterations in the expression of MAPK family proteins: p-JNK ↓, p-p38 ↓, p-ERK ½ ↓,	[117]
IEC-6 rat intestinal epithelial cells with colitis model; Caco2 human colorectal cancer cells	In vivo bromelain treatment + 2,4,6-trinitrobenzene sulfonic acid (TNBS) In vitro bromelain	Anti-inflammatory activity: transmembrane tumor necrosis factor receptors ½ (TNFR1/2) ↓, NF-κB ↓, Bax ↓, Bcl-2 ↑	[122]
C57BL/6 IL-10-deficient mice with spontaneous colitis	In vitro bromelain treatment	Proteolytic degradation of several cell surface molecules: CD44 ↓, CD45R ↓, CD62L ↓, CD8 ↓ Reduction the the clinical and histologic severity of IBD	[123,125]
Human cells from endoscopic colon biopsies from patients with ulcerative colitis, Crohn’s disease	In vitro bromelain treatment	Anti-inflammatory activity: granulocyte colony stimulating factor (G-CSF) ↓, granulocyte-macrophage colony stimulating factor (GM-CSF) ↓, IFN-γ ↓, CCL4/macrophage inhibitory protein (MIP)-1β ↓, TNF-α ↓	[124]
PBMC human peripheral blood mononuclear cells	In vitro bromelain treatment In vivo bromelain treatment	Anti-inflammatory activity: CD7 ↓, CD8α ↓, CD14 ↓, CD16 ↓, CD21 ↓, CD25 ↓, CD41 ↓, CD42a ↓, CD44 ↓, CD45RA ↓, CD48 ↓, CD57 ↓, CD62L ↓, CD128a ↓, CD128b ↓, CD128a/CXCR1 ↓, CD128b/CXCR2 ↓	[127,128,129,130]
Ovalbumin (OVA)-induced murine model of allergic airway disease	In vitro bromelain treatment	Inhibition allergic sensitization: CD4+ ↓ CD8+ ↓, CD4+↓, CD25+ ↓, CD44 ↓	[134,135]

The effects of bromelain are marked as follows: ↓ decreased, ↑ increased.

## 7. Anticancer Properties of Bromelain

### 7.1. Apoptosis, Cell Cycle Arrest, and Cell Survival

**Figure 1:**
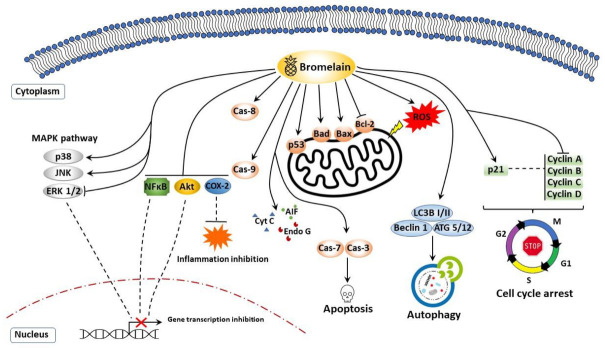
Possible molecular mechanisms of bromelain’s anti-tumor activity are realized at 3 levels of cellular metabolism. In vitro and in vivo studies have shown that bromelain inhibits the proliferation of cancer cells primarily by I) modulating the expression of genes crucial for cell differentiation and proliferation (MAPK signaling pathway, Akt, Cox-2, NF-κB), II) induction of cell death by apoptosis/autophagy, and III) blocking the cell cycle by inhibiting cyclins which are necessary for this process.

Unfortunately, the existing clinical trials using bromelain in cancer treatment provide little information on its antitumor activity. However, new in vitro and in vivo studies using bromelain as a potential chemotherapeutic agent constantly appear. It is worth noting that the exact mechanism of its molecular anticancer activities is still unknown. It has been suggested that the ability of bromelain to inhibit tumor cell proliferation and metastasis, and induce tumor cell death may be due to its proteolytic and immunomodulatory properties. Bromelain’s ability to trigger apoptosis is undoubtedly one of its essential features allowing for effective inhibition of cancer development and proliferation. Considerable evidence suggests that bromelain as a potential promising anticancer agent affects the expression of many genes involved in programmed cell death, proliferation, or metastasis. As shown in Figure 1, the molecular mechanisms of bromelain’s anticancer activity are carried out in many biochemical pathways. The therapeutic potential of bromelain and its efficacy in activating PCD processes and modulating the expression of apoptosis-associated proteins has been investigated at molecular levels in many in vitro and in vivo cancer models involving breast cancer [136,137,138], ovarian, lung, melanoma [139,140,141], digestive system cancer [142,143,144,145,146,147], and leukemia [148,149,150]. Table 2 shows the most important molecular mechanisms of bromelain’s anti-tumor activity.

#### 7.1.1. Breast Cancer

Bromelain was found to have effective anticancer activity against breast cancer cells—MCF7, MDA-MB231, and GI-101A. Bromelain not only inhibited tumor cell proliferation and colony formation but also influenced several critical biochemical processes, ultimately leading to their death by apoptosis/autophagy [136,137,138]. Excellent information on the complexity of molecular activities of bromelain in modulating the expression of key genes, including proliferation, migration, and DNA integrity, was provided by the studies of Fouz et al. [151]. Undoubtedly, the primary mechanism of action of bromelain in breast cancer cells is the induction of apoptosis. Programmed cell death is carried out mainly by the mitochondrial route, with a bromelain-dependent increase in the activity of caspases 3 and 9. Analysis of gene expression showed that bromelain also increased the activity of proapoptotic proteins from the Bcl-2 family, with a simultaneous decrease in the expression of their anti-apoptotic partners. At the same time, in addition to induction of cancer cell death, enzymatic degradation of cell DNA and inhibition of their cell cycle in the G2/M phase was observed due to activation of cell cycle inhibitors [136,137,138]. Interesting information on the effectiveness of the anticancer activity of bromelain was provided by the studies of Bhatnagar et al. [152]. Encapsulation of bromelain in Poly (lactic-co-glycolic acid) (PLGA) to formulate nanoparticles increased its bioavailability, cellular uptake, and total anticancer potential.

#### 7.1.2. Melanoma and Epidermoid Carcinoma

Studies using murine and human melanoma and epidermal carcinoma cell models confirmed the high antitumor potential of bromelain [139,140,141]. It was shown that treatment with bromelain resulted in the upregulation of key genes for apoptosis and the cell cycle, including the p53 suppressor. Inhibition of G2/M phase cell division by bromelain was mediated by modulation of cyclin B1. Bromelain-dependent apoptosis induction was associated with modulation of the Bax-Bcl-2 ratio, activation of caspases 3 and 9, and DNA fragmentation. As indicated by Bhui et al. [140], bromelain’s ability to deplete intracellular glutathione and generation of reactive oxygen species also plays an essential role in shaping the bromelain’s antitumor properties against melanoma and epidermal cancer.

Significantly, bromelain not only reduced the proliferation of skin cancer cells and led to their death by apoptosis but also caused a marked inhibition of cyclooxygenase-2 (COX-2) expression and inactivation of the NF-κB by blocking phosphorylation and subsequent degradation of nuclear factor of kappa light polypeptide gene enhancer in B cells inhibitor (IκBα). As over-induction of COX-2 is often involved in tumorigenesis and is regulated by NF-κB, bromelain-dependent inactivation of these genes is likely responsible for its molecular antitumor mechanisms. In addition, treatment with bromelain significantly reduced the transduction of extracellular signal-regulated protein kinase (ERK1/2), p38 mitogen-activated protein kinase MAPK, and Akt activity, whose enhanced activity correlates with tumor growth [139,141].

#### 7.1.3. Chronic Myelogenous Leukemia and Lymphomas

Studies using bromelain against murine leukemia models and human chronic myelogenous leukemia indicate that combining bromelain with pineapple peroxidase is more effective in inhibiting tumor progression than bromelain alone. Thus, the use of bromelain plus pineapple peroxidase may be a powerful natural anticancer agent. The combination of bromelain-peroxidase caused inhibition of the cell cycle in the G0/G1 phase, and increased intracellular reactive oxygen species (ROS) levels and changes in mitochondrial potential. The induction of the mitochondrial apoptotic pathway and the increase in the level of cytochrome C, Bad, and Bax, in addition to the reduction in Bcl-2 and NF-κB, was again crucial for the antitumor properties of bromelain to leukemic cells [148,149,150].

#### 7.1.4. Cancers of the Digestive System—Gastrointestinal and Colon Cancer

The complexity of the antitumor mechanisms of bromelain concerning the various types of gastrointestinal cancers is remarkable. Research shows that bromelain used alone or in combination therapy with N-acetylcysteine effectively inhibited the proliferation of human gastric and colon carcinoma cells [142,143,144,145,146]. Moreover, as Romano et al. [147] reported, in vivo, the bromelain reduced the development of aberrant crypt foci, polyps, and tumors induced by azoxymethane. 

The biological activity of bromelain has been associated with various mechanisms of cell death. The apoptogenic properties of bromelain were realized in the mitochondrial pathway and were associated with the involvement of the caspase system and extranuclear p53, poly (ADP-ribose) polymerase (PARP) degradation. Bromelain also appears to impair cancer cell survival by blocking the ERK1/2 and pAkt/Akt, NFκB/MAPK pathway and attenuating Bcl-2 and mucin 1, cell surface associated (MUC1) oncoproteins [142,143,144,147]. It is worth emphasizing that the MUC1 glycoprotein, which provides tumor cells with invasive, metastatic, and chemo-resistant properties, may be one of the main targets of bromelain’s antitumor activity [153].

In addition to inducing apoptosis, bromelain induces autophagy in gastrointestinal cancer cells. Gene expression analysis studies have shown a bromelain-dependent increase in genes involved in this form of cell death—including Atg3, Atg5, Atg7, Atg12, Beclin 1, p62, and LC3 conversion [143,145].

One of the latest studies conducted by the research team of Park et al. [146] provided valuable information on the anticancer mechanisms of bromelain’s activity, pointing to another mechanism of its action. Studies have shown that bromelain in Kras-mutant colorectal cancer caused a remarkably elevated expression of ACSL-4, which has been suggested to play a crucial role in regulating ferroptosis and sensitizing cells to this type of cell death. As emphasized by the authors, bromelain induces ROS-induced ferroptosis in Kras mutant CRC cells via ACSL-4, proving bromelain’s role in another pathway of cancer cell death.

#### 7.1.5. Hepatic and Pancreatic Cancer

Due to the low penetration capacity of the available chemotherapeutic agents of the pancreatic organ as the result of the dense extracellular matrix, treating cancer of this organ is one of the most challenging tasks for modern chemotherapy. There is a high promise for bromelain, which can proteolytically degrade the extracellular matrix (ECM). However, due to its short blood half-life, it is unable to accumulate sufficiently in the pancreas. Recent research indicates the development of novel, effective delivery methods of anticancer drugs in treating pancreatic cancer using supramolecular glycosylated polyethylene bromelain. It is worth noting that in such a biological system, bromelain does not directly act against cancer. The role of Polyethylene-Glycosylated bromelain is enzymatic degradation of the ECM around the tumor, which results in the enhancement of the penetration of anticancer drugs into the neoplastic tissue of the pancreas. As an anticancer drug delivery system, bromelain plays the role of a specific enhancer of the activity of chemotherapeutic agents [154].

Another application of the combination of bromelain and N-acetylcysteine in the treatment of liver and pancreatic tumors was proposed by Pillai et al. [155]. The synergistic action of the mixture of these compounds with commercially available chemotherapeutic agents such as doxorubicin, cisplatin, 5-Fluorouracil (5-FU) in liver/triplet tumor cells has been demonstrated. These properties may significantly reduce the doses of chemotherapeutic agents while increasing the effectiveness and frequency of anticancer therapy with minimized effects of systemic toxicity.

#### 7.1.6. Other Strategies in Bromelain-Dependent Cancer Treatment

On the basis of the studies cited above, bromelain undoubtedly appears to be a potent inducer of apoptosis and autophagy in tumor cells. The increase in the expression of key genes for the PCD mitochondrial pathway, autophagy, or cell cycle inhibition observed during bromelain treatment proves its strong anticancer properties while indicating that these are the mechanisms responsible for shaping its biological properties against various types of cancers. It is worth noting that several reports indicate that bromelain is effective in inhibiting the development of other types of cancer—soft tissue sarcoma [156], lung [152], and oral cancer cells [157]. An innovative application of bromelain in available chemotherapy was proposed by Mekkawy et al. [158]. The radiosensitizing and radioprotective efficacy of bromelain was investigated in in vitro and in vivo studies. Bromelain possessed a dual function due to its simultaneous sensitization of cancer cells and protection of normal cells during radiotherapy. The authors suggest that bromelain can be considered a radiosensitizer and radioprotector, suggesting a possible role in dose reduction during radiotherapy.

### 7.2. Autophagy

Autophagy is an evolutionarily conservative process of intracellular digestion of cytoplasmic macromolecules and organelles in lysosomes, which is involved in maintaining cellular homeostasis under both optimal growth conditions and keeping the cell alive under stressful conditions. The role of autophagy is to degrade unnecessary or damaged elements of cells that are extremely important in many human diseases, especially cancer. Based on previous literature reports, the role of autophagy in the process of carcinogenesis cannot be clearly assessed. Autophagy may function as a mechanism for the survival of metabolically altered cancer cells, but most of the results obtained show autophagy as a mechanism that prevents cancer development. In most cases, an increase in the expression of proteins involved in the induction of autophagy simultaneously inhibits tumor transformation [159].

Several reports indicate that, in addition to induction of apoptosis in cancer cells, bromelain can trigger autophagy, preceding cell death and inhibition of carcinogenesis [138,143,145]. Bromelain treatment of two breast cancer cell lines (positive estrogen receptor MCF-7 and negative MDA-MB-231) inhibited both cell line proliferation in a time- and dose-dependent manner. The autophagy-inducing potential of bromelain has been confirmed in the kinase expression studies of the MAPK pathway. MAPK protein family regulates many intracellular processes, including gene transcription, protein biosynthesis, cell division, differentiation, and survival or apoptosis/autophagy. It has been shown that bromelain treatment results in an upregulation in the expression of c-jun N-terminal kinase (JNK) and p38 kinase, which are mainly involved in cell death, whereas phosphorylation of extracellular signal-regulated kinase 1/2 (ERK1/2) was downregulated.

Furthermore, bromelain was seen to induce the expressions of autophagy-related proteins, light chain 3 protein B II (LC3BII), and beclin-1. Flow cytometry analysis showed that bromelain stimulates apoptosis following autophagy led to an increase in the population of Sub-G1 cells [138]. Combination treatment of human gastrointestinal carcinoma cells lines, including MKN45, KATO-III, HT29-5F12, HT29-5M21, and LS174T using bromelain and N-acetylcysteine also provided relevant information about the anticancer properties of bromelain. The combination of bromelain and N-acetylcysteine caused cell cycle inhibition (decreased level of cyclins A, B, and D), and induced cell death with involvement of both apoptotic and autophagic processes. After combination therapy, the emergence of the autophagosome marker LC3-II and expression changes of other autophagy-related proteins, including Atg3, Atg5, Atg7, Atg12, and beclin 1, were observed [143]. These results align with the latest research by Chang et al. [145], which confirms bromelain’s antitumor abilities in colorectal cancer cells (CRC). Bromelain treatment suppresses CRC progression through, among others factors, modulating autophagy-related gene expressions, resulting in autophagy and lysosome formation. Cheng et al. postulate that bromelain-dependent autophagy induction results from an increase in the activity of crucial proteins for this process, such as ATG5/12, beclin 1, and p62. In addition, an increase in the conversion of LC3-I to LC3-II after bromelain treatment was observed as crucial for autophagosome formation.

### 7.3. Chemosensitizing Effect of a Combination of Bromelain

Effective chemotherapy characterized by low systemic cytotoxicity and lack of undesirable side effects of treatment is a great challenge for modern medicine. Commercially used drugs such as cisplatin or doxorubicin are characterized by good antitumor efficacy. However, their use is often fraught with adverse side effects such as neurotoxicity, nephrotoxicity, myelosuppression, and ototoxicity, affecting patients’ general health. Many commercial chemotherapeutic agents are currently administered in combination therapy with other drugs, adjuvants, or monoclonal antibodies [160]. Plant compounds are also increasingly popular, as they often have high anticancer potential and protective effects on the body. Combining compounds of plant origin with commercial chemotherapeutics can be an effective strategy to minimize the severity of side effects associated with individual drugs while maintaining or even increasing the effectiveness of the treatment process [161].

The positive effects of using bromelain as a factor supporting conventional chemotherapy and increasing its effectiveness have been confirmed in several in vitro and in vivo studies. The combination of idarubicin and bromelain significantly reduced HL-60 tumor cell proliferation mainly by triggering apoptosis pathways and genotoxic properties. As the authors indicate, idarubicin combined with bromelain produces more cytotoxic effects in low concentrations than as a single drug administrated against HL-60. It is also worth emphasizing that this combination in PBMC resulted in less cytotoxicity and genotoxicity in comparison to tumor cells [162].

The synergistic effect of bromelain with another chemotherapeutic agent—cisplatin—has been confirmed in a number of studies using a breast cancer model [163,164,165], human prostatic [166], and gastric [165] carcinoma, and peritoneal mesothelioma cells [153,167]. Synergistic enhancement of the antitumor effect was observed in MD-MB-231 and MCF7 cells treated with the combination of bromelain and cisplatin compared to bromelain or cisplatin single treatment. Bromelain-cisplatin combination treatment resulted in the enhanced activation of apoptosis on the mitochondrial pathway. A decrease in expression of anti-apoptotic proteins such as Bcl-x and HSP70 was observed with a simultaneous increase in the level of their proapoptotic antagonist Bax [164,165]. The synergism of the antitumor activity of the bromelain-cisplatin combination has also been confirmed in vivo in 4T1 breast tumor model studies. The obtained results indicate that bromelain treatment may increase the antitumor effect of cisplatin on triple-negative 4T1 breast cancer cells through modulating the inflammation in the tumor environment. The use of the combination resulted in a significant reduction in the size of tumor and lung metastases. Moreover, combination treatment showed downregulation of the expression of inflammation-related genes that are involved in tumor inflammation, such as Gremlin (GREM1), IL-1β, IL-4, NF-κB, and prostaglandin-endoperoxide synthase 2 (PTGS2), tumor nitric oxide level [163]. In studies using human prostatic carcinoma cells, it was shown that the combination of cisplatin with bromelain resulted in a significant increase in the antitumor activity of the chemotherapeutic. Cisplatin-bromelain showed a synergistic antitumor effect in vitro by inhibiting colony formation, in addition to increased p53 proapoptotic gene expression. Notably, the use of bromelain significantly allowed reduction in the required dose of cisplatin [166]. Similar synergistic effects were observed when using the bromelain-cisplatin combination against malignant peritoneal mesothelioma cells. Combination treatment significantly increased the antitumor activity of cisplatin. A decrease/increase in expression of many genes involved in proliferation and apoptosis, respectively, was observed [153,167].

However, it is worth emphasizing that, as shown by the research of Raeisi et al. [165], the occurrence of the synergistic effect of the anticancer effect of bromelain and commercial chemotherapeutic agents may depend on the dose of the drug used and the type of cancer. The authors emphasize that bromelain combined with cisplatin showed a synergistic effect on MCF7 cells while being additive or antagonistic with cisplatin inhibitory concentration (IC) IC_30_ and IC_40_ to AGS cell proliferation. Moreover, the combination of bromelain with 5-FU worked quite differently, showing a synergistic effect on AGS cells while being antagonistic to MCF7 cells. Similar conclusions were drawn from studies using pancreatic and hepatic cancer cells. The enhancement of the action of chemotherapeutic agents in combination with bromelain was concentration-dependent and cell line-specific. It is worth noting that bromelain showed a synergistic effect with a wide range of chemotherapeutic agents [163]—gemcitabine, 5-FU, doxorubicin, and cisplatin [155].

As indicated by the latest research by Wang et al. [168], bromelain in combined anticancer therapy with a commercially available chemotherapeutic drug can also be carried out using the latest nanotechnology effects. Bromelain produces synergistic antitumor effects with doxorubicine. The use of bromelain nanoparticles as a carrier for doxorubicin increased drug antitumor activity. Doxorubicine/bromelain nanoparticles displayed a higher permeation profile. In addition, it was demonstrated in in vivo experiments that these carriers can transfer more doxorubicin to the tumor site in contrast to the drug used alone. As the authors emphasize that bromelain nanoparticles can be potential drug carriers for efficient drug delivery.

### 7.4. Reactive Oxygen Species (ROS) 

Reactive oxygen species are an inseparable element of our cellular metabolism that plays a vital role in the proper functioning of many cellular processes. The homeostasis disorder between ROS production and antioxidant systems leads to oxidative stress, damaging cellular macromolecules, i.e., DNA, proteins, lipids, and subsequently, tumor transformation. Due to the duality of the biological activity of ROS against cancer cells and the development of the tumor formation process, it is challenging to attribute negative or positive effects to them. The high concentration of ROS is characteristic of the initial stages of carcinogenesis and clonal expansion. It increases the proliferation of cancer cells and promotes angiogenesis, the formation of further mutations in DNA, and further reprogramming of cell metabolism.

However, more and more studies indicate the use of compounds in anticancer therapy that inhibit mechanisms that prevent or induce oxidative stress by generating reactive oxygen species in cancer cells favors their apoptosis and reduces tumor size. The role of ROS in the antitumor activity of many commonly used chemotherapeutics, including paclitaxel, docetaxel, cisplatin, or doxorubicin, has already been documented. Many natural compounds can inhibit the activity of antioxidant enzymes with simultaneous pro-oxidative properties [169,170,171]. However, great caution should be exercised when evaluating the anticancer activity of natural compounds resulting from their pro-oxidative properties.

One of the first studies on the pro-oxidative properties of bromelain was conducted using human polymorphonuclear neutrophils from healthy donors and human melanoma cell line, Sk-mel28 [172]. It has been shown that bromelain is able to stimulate the innate immune system by activating neutrophils to produce ROS, which, as the authors emphasize, is presumably one of the mechanisms of its antitumor activity. Brakebusch et al. [96] also report ROS production by bromelain in granulocytes and monocytes in healthy volunteers and patients with disorders of the humoral immune system. The ability of bromelain to change the intracellular level of ROS would directly affect signaling modulation in both immune and cancer cells. 

The pro-oxidative properties of bromelain against cancer have also been confirmed in later studies. Bromelain treatment resulted in depletion of intracellular glutathione (GSH) content and a simultaneous increase in ROS production in A431 epidermal and A375 melanoma cancer cells. It is suggested that bromelain-dependent depletion of GSH may be attributed to its cysteine protease activity. The consequence of the ROS increase and the generation of oxidative stress by bromelain in A431/A375 was ROS-mediated cell cycle arrest in the G_2_/M phase, mitochondrial potential impairment, and, ultimately, apoptosis [140]. Chang et al. [145] proved that exposure of HCT116 and HT-29 cells to bromelain resulted in significant oxidative stress and superoxide production induction. Excessive concentration of ROS formed was the cause of the initiation of autophagy first, followed by irreversible damage to cancer cells and their final death on the apoptosis pathway.

## 8. Potential Clinical Applications of Bromelain 

Due to the health-promoting biological properties and high complexity of the molecular mechanisms of activity, bromelain is used in the treatment of various diseases. As clinical research indicates, bromelain is used with significant effect alone or in combination with other nutraceutical preparations (most often with trypsin and routine) in many areas of medicine [6,23,40,43]. Here we present a brief overview of the most essential clinical and pharmaceutical applications of bromelain.

### 8.1. Allergic Sensitization 

As demonstrated by Secor et al. [134,135], due to its immunomodulatory properties, bromelain can be used to inhibit allergic sensitization and murine asthma. It has been shown that bromelain can limit the development of allergic airway disease through the inhibition/modulation of a critical component of the allergic airway disease response, such as the influx of lymphocytes and eosinophils into the lung, reduction in CD4+, CD8+, and CD4+ CD25+ T lymphocytes. Additionally, bromelain prevented allergic sensitization by proteolytic degradation of CD44, a crucial surface marker in dendritic cell activation of T cells.

### 8.2. Chronic Rhinosinusitis

Chronic rhinosinusitis is currently one of the most common long-term respiratory diseases. Due to the poorly understood pathophysiology of the disease, an effective treatment is a current challenge for medicine [173,174]. Bromelain is considered an attractive candidate in therapy against chronic rhinosinusitis [175,176]. It is believed that bromelain may reduce the secretion of pro-inflammatory agents during rhinitis and mucus secretion and aids its drainage [45,174]. Matschke et al. [177] emphasize the therapeutic use and benefits of postoperative use of bromelain in otorhinolaryngology. As the authors note, the anti-oedema effect of bromelain was the most significant benefit during the postoperative convalescence period. A decrease in the severity of pain, swelling, or signs of inflammation was observed. According to Passali et al. [178], an appropriate bromelain’s biological activity level may be due to its excellent distribution by diffusion from the blood to rhinosinusal mucosa.

### 8.3. Enzymatic Debridement and Pro-Wound Healing Activities

Bromelain-based debridement has been proven to be an efficient enzymatic debridement agent [179,180,181,182]. Protease activity of bromelain contributes to the degradation of collagen, elastin, laminin, fibronectin, and other damaged extracellular matrix components. One of the components of bromelain is escharase, which has no proteolytic properties, and is likely responsible for the protective effect of bromelain on the skin with burn wounds and the effective elimination of eschar [183].

The mechanisms responsible for effective wound debridement and removal of necrotic cells by bromelain include its influence on modulating the expression of factors involved in pro- and anti-inflammatory processes, such as TNF-α, transforming growth factor beta (TGF-β) [184]. Ghensi et al. [185] notes that the pro-wound healing activities of bromelain results from its activation of mesenchymal stem cells and an increase in the anti-inflammatory activity of IL-10.

To date, numerous studies have been conducted to increase the stability of bromelain and its preparations and to improve the activity in the treatment of burns. [186]. It is worth noting that Schulz et al. [187] and Berner et al. [188] pay attention to the low selectivity of the bromelain-based enzymatic debridement (NexoBrid^®^), which has a toxic effect on viable human skin cells, especially at high concentrations.

### 8.4. Osteoarthritis

Osteoarthritis (OA) is the most common musculoskeletal, degenerative disorder characterized by the chronic inflammatory response, clinically manifested by joint pain and reduced mobility. Commonly used to treat osteoarthritis are anti-inflammatory drugs [189]. Several studies have been performed on OA treatments indicating that bromelain may be an attractive method for dealing with this disease due to its potent analgesic and anti-inflammatory activity. Bromelain contributes to decreasing oxidative stress and the expression of inflammatory mediators, including bradykinin, thromboxane A2, and PGE_2_ [190,191]. Recent studies prove that using bromelain in combination therapy with other nutraceuticals, such as diclofenac sodium, rutin, trypsin, or turmeric, results in significantly enhanced efficacy in the treatment of degenerative joint pain diseases [191,192,193,194].

### 8.5. Musculoskeletal Function and Surgical Procedures 

Several studies indicate that bromelain reduces swelling and pain resulting from injuries or muscle damage [195]. In animal studies, Aiyegbusi et al. [196] showed a significant bromelain-dependent increase in the tenoblast proliferation and, thus, healing progression by stimulating the tenocyte population. Several human studies also suggest similar benefits. Bromelain, in combination with fungal proteases and papain, improves muscle function after eccentric exercise. Protease supplementation attenuates muscle strength losses after eccentric exercise by regulating leukocyte activity and inflammation and allow for faster regeneration of muscles [197,198]. 

## 9. Bromelain as a Possible Treatment for COVID-19 Disease

### 9.1. COVID-19 Disease

Coronavirus disease 2019 (COVID-19) is caused by severe acute respiratory syndrome coronavirus-2 (SARS-CoV-2). The initial outbreak of the disease was reported in a market in Wuhan (Hubei Province, China) in December 2019. Since then, the disease has expanded globally, reaching pandemic proportions [199]. According to WHO reports, at the beginning of November 2021, there had been over 240 million confirmed cases of COVID-19, including over 5 million deaths worldwide [200]. Despite the introduction of the COVID-19 vaccine, the number of infected patients has continued to increase. This situation results from a lack of specific and effective treatment, delayed widespread availability of the COVID-19 vaccine, and differences in public trust in COVID-19 vaccines. Moreover, the length of immune protection after vaccination may be limited, and novel SARS-CoV-2 variants may reduce the efficacy of the vaccines. Publication of the genetic sequence of COVID-19 in January 2020 facilitated the swift development of testing kits and accelerated the global race to vaccinate. Despite the heroic efforts of experts with diverse backgrounds and compressed schedules that shortened the standard vaccine development timeline, it was found that the development of COVID-19 vaccines was not the only enormous challenge. The other problems in vaccinating most of the global population are gaining and maintaining public trust in COVID-19 vaccines and vaccination, the scarce resources of the COVID-19 vaccine, and its delayed widespread availability. As of the beginning of November 2021, a total of 7 billion of vaccine doses have been administered. Unfortunately, the number of infected patients has continued to increase. Additionally, it was confirmed that the length of immune protection against SARS-CoV-2 virus after vaccination may be limited [201,202].

Thus, at the same time, diverse trials are currently running worldwide [203] to discover effective drugs to prevent the infection, eradicate the virus, and manage disease complications. Hopefully, a few therapeutic agents will be proven to reduce early- and late-stage disease progression [204]. Patients with COVID-19 are treated with antiviral drugs, viral protease inhibitors, anti-inflammatory agents, antimalarials, nucleoside analogues, neuraminidase inhibitors, DNA synthesis inhibitors, ACE2-based peptides, novel vinylsulfone protease inhibitors, teicoplanin, and 3-chymotrypsin-like protease and papain-like protease inhibitors [205,206,207]. However, there are still concerns about the efficacy of these drugs due to a lack of valid clinical trial data. 

Thus, the continued exploration of effective treatments is still needed. Researchers have involved alternative therapies based on natural medicinal products due to their possible antiviral and anti-inflammatory activity [208]. Natural substances exhibit antiviral capabilities against already known viral pathogens such as coronavirus SARS-CoV, coxsackievirus, hepatitis B virus, hepatitis C virus, herpes simplex virus (HSV), human immunodeficiency virus (HIV), influenza virus, and respiratory syncytial virus (RSV) [209]. However, these findings mostly derive from laboratory studies, whereas clinical data are limited. It is supposed that phytotherapeutics may fight infections caused by coronaviruses in two ways, by general improving the immune system viral penetration and replication [210].

The pathogen responsible for the pandemic belongs to the subfamily Coronavirinae in the family Coronaviridae of the order Nidovirales. Two other highly pathogenic ß-coronaviruses have already been identified in humans—SARS-CoV and the Middle East Acute Respiratory Syndrome Coronavirus (MERS-CoV), which causes severe types of lower respiratory tract infection and acute respiratory distress syndromes (ARDS) [211]. SARS-CoV-2 is a novel β-coronavirus, with either a round or elliptical form with an approximate diameter of 60–140 nm [208]. It is an enveloped RNA virus with positive polarity and a single-strand RNA genome comprised of 30 kb. It encodes in its genome at least 29 proteins, four of which are structural: the spike (S), membrane (M), envelope (E), and nucleocapsid (N) proteins [212]. The virus rapidly spreads via airborne transmission from human to human, but genetic evidence suggests it is a natural virus that originated in animals. Unfortunately, the knowledge about the animal origin of SARS-CoV-2 and how the virus has passed from the animal host to the human remains incomplete [213]. The disease spreads mainly by fomites and droplets during close unprotected contact between the infected and uninfected. The virus may also be spread indirectly by virus-containing respiratory droplets, which contaminate people’s hands and finally contact the mucous membranes of the mouth, nose, and eyes, causing infection [214].

The first symptoms of the disease, such as fever, dry cough, and fatigue, appear after an incubation period of around 2–14 days. The majority of patients develop mild to moderate illness and recover without hospitalization. A pathogenic virus targets mainly the human respiratory system. However, it also may lead to loss of taste or smell, a rash on the skin, discoloration of fingers or toes, myocardial injury, arrhythmic complications, neurological complications, gastrointestinal disorders, and musculoskeletal symptoms. Patients with fatal disease have quickly developed acute respiratory distress syndrome (ARDS), hard-to-correct metabolic acidosis, septic shock, and coagulation dysfunction, and died of multiple organ functional failure [205,215]. The COVID-19 disease symptoms differ with age. Elderly patients (older than 60 years) and those with underlying diseases have a worse prognosis because of a greater chance of respiratory failure and longer disease courses [213].

### 9.2. Possible Management of Bromelain in COVID-19 Disease Treatment

The coronavirus-2 (SARS-CoV-2) viral entry is crucial for viral replication and is an ideal therapeutic target. The virus infects the host cell using receptor-mediated endocytosis via the membrane-bound aminopeptidase angiotensin-converting enzyme II receptor (ACE2) [216]. The SARS-CoV-2 attaches to the host receptor ACE2 by the spike protein (S), a heavily glycosylated type I transmembrane glycoprotein [217]. However, the virus needs proteolytic processing of the S protein to activate the endocytic route. It has been shown that host proteases participate in the cleavage of the S protein and activate the entry of SARS-CoV-2, including transmembrane serine protease 2 (TMPRSS2), cathepsin L (CTSL), and furin [215,218,219]. These proteases induce a significant structural rearrangement of the receptor-binding domain (RBD) of the spike protein (S), which triggers the transition from the pre-fusion to the post-fusion state [220,221]. The angiotensin-converting-enzyme II receptor (ACE2) is expressed in various organs such as the lung, heart, kidney, intestine, gall bladder, and testicular tissues. Moreover, TMPRSS2 is highly expressed in several tissues and body sites and is co-expressed with ACE2 in nasal epithelial cells, lungs, and bronchial branches; this explains some tissue tropism of SARS-CoV-2 [222,223]. Because the density of the ACE2 is higher in adults than in children, the predisposition to infection and severe disease grows with age [224].

ACE2 and TMPRSS2 contain cysteine residues with disulfide bonds to stabilize the protein structure [225,226]. Moreover, the spike glycoprotein and envelope protein of SARS-CoV-2 contain disulfide bridges for stabilization, and they are essential for binding to the ACE2 receptor in host cells [220,227]. Thus, disulfide bonds represent an attractive target for bromelain, a cysteine protease capable of breaking disulfide bonds and degrading proteins. Sagar et al. (2021) [226] found that bromelain can inhibit SARS-CoV-2 infection via targeting ACE2, TMPRSS2, and SARS-CoV-2 S-protein. Bromelain induced a dose- and time-dependent reduction in ACE2 and TMPRSS2 expression in VeroE6 cells. Bromelain’s cysteine protease activity was responsible not only for the cleavage of host cells’ ACE2 but also SARS-CoV-2 S-protein. Akhter et al. [228] found that bromelain used alone (50 and 100 µg/mL) and in combination with acetylcysteine (50 and 100 µg/20 mg/mL) can disrupt the integrity of spike and envelope proteins of the SARS-CoV-2 virus. Bromelain pre-treatment significantly blocked SARS-CoV-2 viral binding in VeroE6 cells and, as a result, reduced the viral infection and SARS-CoV-2 viral RNA copies inside the cells [226]. Similarly, when bromelain’s multipotent enzymatic competencies were complemented with acetylcysteine’s strong power to reduce disulfide bonds, inhibition of the infectivity of SARS-CoV-2 was observed [228]. 

Tallei et al. [229], for the first time, applied molecular docking and molecular dynamics simulation approaches to investigate the ability of bromelain to counteract various variants of the SARS-CoV-2 by targeting bromelain binding on the side of this viral interaction with angiotensin converting enzyme 2 (ACE2). The researchers found that bromelain exhibited good binding affinity toward various variants of the receptor-binding domain (RBD) responsible for the attachment of the virus to ACE2. Furthermore, the authors hypothesize that interference by bromelain interaction between RBD and hACE2 enables its potential use for prevention against viral entry into the host cells. This study lays the groundwork for the further in vitro and in vivo testing that is necessary before any final conclusion can be made.

However, it was already indicated that bromelain can remove the spike and hemagglutinin proteins of Semliki Forest virus, Sindbis virus, mouse gastrointestinal coronavirus, hemagglutinating encephalomyelitis virus, and H1N1 influenza virus [230,231]. It is noteworthy that there are similarities in the structural conformation of the spike protein among human-infecting coronaviruses (mainly between SARS-CoV and SARS-CoV-2) [232]. Thus, it is supposed that bromelain may be used as a broad antiviral agent against SARS-CoV-2 and other related family members [226]. 

The binding of SARS-CoV-2 to the angiotensin-converting enzyme 2 (ACE2) receptor initiates three main pathways: (1) the inflammatory, (2) the coagulation, and (3) the bradykinin cascades [233,234]. The activation of these pathways leads to a wide range of clinical manifestations of COVID-19, from asymptomatic to severe acute respiratory distress syndrome, multiple organ failure, and death [233].

The development of inflammation leads to the massive activation of both neutrophils, dendritic cells, and macrophages which are mobilized to the site of infection. This first line of defense cells increases the production of chemokines and cytokines that promote the onset of inflammation (cytokine storm). Patients with severe SARS-CoV-2 infection present high levels of pro-inflammatory cytokines such as IL-1β, IL-2, IL-6, IL-7, IL-8, IL-9, IL-10, and TNFα, and chemokines such as GCSF, IP-10, MCP-1, and MIP-1α, in the plasma. Most patients also develop marked lymphopenia, their T-cell count is reduced significantly, and the surviving T-cells appear to be functionally exhausted [211,213]. Cytokine storm syndrome causes acute respiratory distress syndrome and respiratory failure, which is considered a leading cause of death in patients with COVID-19. In experimental studies, bromelain presents unique immunomodulatory actions. Bromelain potentially activates the healthy immune system in response to cellular stress by activation of the release of inflammatory mediators, including interleukin IL-1β, IL-6, interferon (INF)-γ and tumor necrosis factor (TNF)-α, modulation of T cell responses and increasing T-cell proliferation. Furthermore, bromelain can inhibit IL-1β, IL-6, and TNF-α secretion when immune cells are already stimulated in the condition of inflammation-induced overproduction of cytokines. Moreover, by downregulation of the pro-inflammatory prostaglandin E-2 (PGE-2) through inhibition of NF-κB and cyclooxygenase 2 (COX-2), bromelain may regulate overexpressed immunogenic pathways and reduce pulmonary inflammation [235]. In the light of data that most of the deaths from COVID-19 are caused by the hyper-inflammatory process, the beneficial effect of bromelain on the immune system seems to be promising in supporting/improving clinical outcomes among COVID-19 patients [236].

As a consequence of increased cytokine levels, a common feature in patients with COVID-19 is coagulation disorders, high venous thromboembolism (VTE), and disseminated intravascular coagulation (DIC) [237]. Preliminary reports on COVID-19 patients’ clinical and laboratory findings indicate thrombocytopenia, elevated D-dimer and fibrinogen levels, prolonged prothrombin time, and disseminated intravascular coagulation [238]. Additionally, early studies have suggested that elevated circulating D-dimer (a product of cross-linked fibrin) levels are associated with mortality among COVID-19 patients [237]. Importantly, bromelain prevents thrombosis formation, thrombophlebitis, and fibrinolytic activities by stimulating the conversion of plasminogen to plasmin and preventing platelet aggregation. Thrombosis and coagulation reduce erythrocyte blood circulation and affect the ventilation-perfusion rate in COVID-19 patients and lead to acute respiratory distress syndrome development [239]. Thus, bromelain’s overall role is to improve cardiovascular and circulatory functions, mainly by diminishing coagulopathy by promoting the free flow of the blood around the circulating system [240].

Pulmonary pathological results suggested that the early pathological changes induced by SARS-CoV-2 pneumonia include pathological interstitial pneumonia and prominent pulmonary oedema, with protein exudation and minor inflammatory cell infiltration. One of the postulated hypotheses explaining the development of pulmonary oedema suggests that suppression of (ACE-2) receptor by SARS-CoV-2 virus may impair the hydrolysis of des-Arg^9^-bradykinin and stimulate the bradykinin receptor type 1 (BKB1) pathway to induce leakage of fluid into the lungs [241,242,243]. Thus, targeting the bradykinin system by either inhibiting bradykinin production or blocking bradykinin receptors may provide new therapeutic options to control COVID-19-induced pulmonary oedema. During animal studies, bromelain displayed the ability to improve inflammation and oedema by hydrolysis bradykinin and reducing kininogen and bradykinin levels in serum and tissues [244]. The ability of bromelain to inhibit the biosynthesis of kinins that promote the development of inflammation may alleviate not only mild symptoms of the COVID-19 (such as cough, fever, swelling, and pain) but also cytokine storm and coagulation disorders, a leading cause of the multiorgan failure [135].

**Figure 2:**
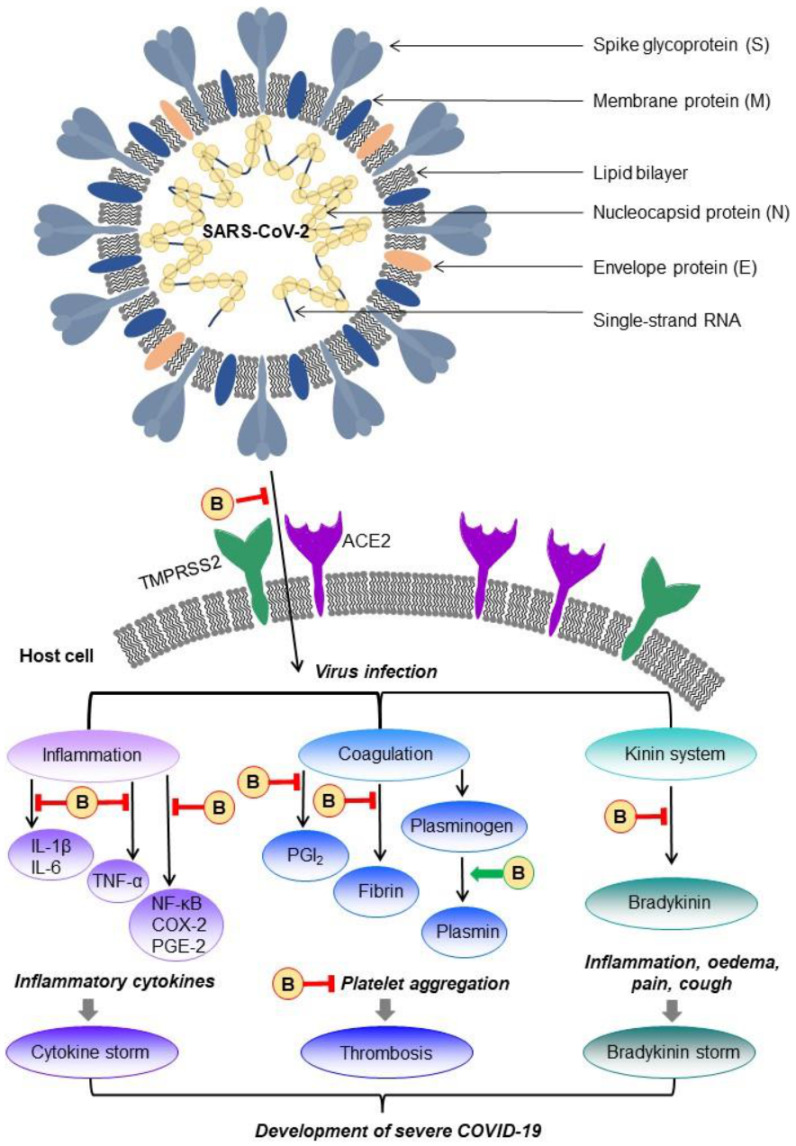
The structure of the SARS-CoV-2 virus and the interference of multiple activity of bromelain (B) in the crucial steps of COVID-19 pathophysiology. TMPRSS2, transmembrane serine protease 2; ACE2, angiotensin-converting enzyme II receptor; IL-1β, interleukin 1 beta; IL-6, interleukin 6; TNF-α, tumor necrosis factor-alpha; NF-κB, nuclear factor kappa-light-chain-enhancer of activated B cells; COX-2, cyclooxygenase 2; PGE-2, prostaglandin E-2; PGI2, prostacyclin.

The drugs being tested for repurposing to treat COVID-19 tend to fall into two categories: those that target the viral replication cycle and those that aim to control the symptoms of the disease. The variety of beneficial effects of bromelain make it one of the potential candidates that could be classified to the second category. Significantly, the anti-inflammatory and anti-coagulatory capacities of bromelain deserve significant scientific attention because they may prevent escalation and the progression of the COVID-19 disease; as shown in Figure 2, bromelain may interfere in the crucial steps of COVID-19 pathophysiology. Additionally, due to its biological properties, bromelain is an attractive compound in treating several CVDs that are common comorbidities with COVID-19 disease [245,246]. The antimicrobial, antifungal, and antibacterial properties seem to be helpful in treating the coinfections in COVID-19 patients. Because bromelain can activate the healthy immune system, it may also support the prevention against SARS-CoV-2 infection, and its rational administration may also become a complementary therapy for COVID-19 and post-COVID-19 patients.

## 10. Conclusions and Future Prospects

Since the mid-1950s, when bromelain first became commercially available, its use has expanded into pharmaceutical, industrial, and clinical applications. Today’s industry draws heavily from benefits offered by bromelain in many fields. Bromelain can be found in many commercially available dietary supplements or cosmetic formulations. The Food and Drug Administration, USA, categorized bromelain as a food additive, and it is among the substances generally accepted as safe.

This study aimed to summarize and highlight the already scientifically established clinical and pharmaceutical applications of bromelain and new reports about its other therapeutic benefits. This review demonstrates that bromelain shows significant promise in medicine and healthcare, especially in treating cardiovascular diseases, blood coagulation and fibrinolysis disorders, infectious diseases, inflammation-associated diseases, and many types of cancer, but the mode of its action is not fully understood. Therefore, there is still an urgent need for studies investigating the mechanism of action and functional properties of bromelain. To date, it has been proven that bromelain is well absorbed in the body after oral administration, and it has no significant side effects, even after prolonged periods. Thus, bromelain can be a promising candidate for the development of oral enzyme therapies for patients. However, the protease is sensitive to high acidity, gastric proteases in the stomach juice, chemicals, organic solvents, and elevated temperature. The instability of bromelain under stress conditions is essential for its practical application because even small conformational changes may reduce protease activity, limiting its health benefits and pharmacological applications. 

Therefore, one of the future tasks for scientists is to overcome bromelain’s stability issues, mainly by devising new techniques and improving existing methods for its stabilization and techniques for its purification. Some of the current investigations are devoted to bromelain stabilization by its immobilization. Enzyme immobilization involves, i.a., nanoparticles of silica, gold, or chitosan. Additionally, bromelain-capped nanoparticles are being investigated as an effective drug delivery carrier of antibiotics or other drugs. 

As a result of the wide range of applications of bromelain, the global demand for bromelain is increasing. However, highly purified commercial bromelain is expensive. In parallel with studies concerning beneficial activities of bromelain, and improvements in its purification and stabilization techniques, many attempts have to be made to develop various strategies to reduce the cost of bromelain production. Interestingly, bromelain extraction from pineapple waste may introduce a significant valorization of the wastes generated by the fruit processing industry, which has already raised some environmental issues. To summarize, combining modernized approaches with novel bromelain production processes is expected to make bromelain production simple, efficient, and economical, leading to the development of stabile, ultrapure, and cheaper bromelain. 

Currently, a matter of great scientific, economic, and medical interest, due to the irrepressible spread of novel variants of the SARS-CoV-2 virus, is to analyze new ways to weaken this virus, prevent its spread, and also eliminate it. Thus, in this review we provided a summary of the available scientific reports concerning the biological properties of bromelain as a potential supportive and prophylaxis treatment of COVD-19 disease. Although bromelain represents an effective compound against the SARS-CoV-2 virus during in vitro or in silico studies, its efficiency has not been tested in vivo using animal and/or human cell models.

In conclusion, bromelain is a biomolecule of great industrial and medical interest; however, there are several areas to be explored in terms of its purification, stabilization, and proper understanding of the mechanism of action, so that the multi-action activities of bromelain can be applied efficiently.

## Figures and Tables

**Table 2:** Cellular and molecular targets of bromelain related to its anticancer activity.

Target	Experimental Approach	Effect	References
GI-101A human breast carcinoma cells	In vitro bromelain treatment	PCD induction: Caspase-3 ↑, Caspase-9 ↑	[136]
AGS human gastric carcinoma cells; MCF7 human breast Adenocarcinoma cells; PC3 human prostate carcinoma cells	In vitro bromelain treatment	Cell proliferative and colony formation inhibition	[137]
MCF7 human breast adenocarcinoma cells; MDA-MB231 human breast cells’ adenocarcinoma triple-negative breast cells	In vitro bromelain treatment	PCD induction: Increase in the population of Sub-G1 cells, alterations in the expression of MAPK family proteins: JNK ↑, p38 ↑, ERK ½ ↓ Autophagy induction: LC3BII ↑, beclin 1 ↑	[138]
HeLa human cervical cancer cells; MCF7 human breast adenocarcinoma cells; A549 human lung carcinoma cells; Caco-2 human epithelial colorectal adenocarcinoma cells; Male, Swiss albino mice	In vitro and in vivo (oral administration) bromelain nanoparticles treatment	PCD induction: Increase in the population of Sub-G1 cells, p53 ↑, Bax ↑, Bcl-2 ↓, ROS ↑ Cell cycle arrest: p21 ↑	[152]
Female, Swiss albino mice—skin tumorigenesis model; A431 human epidermoid carcinoma cells; A375 human melanoma cells;	In vivo (oral administration) bromelain treatment In vitro bromelain treatment	PCD induction: Increase in the population of Sub-G1 cells, p53 ↑, Bax ↑, Bcl-2 ↓, Caspase-3 ↑, Caspase-9 ↑, COX-2 ↓, NF-κB ↓, ERK ½ ↓, p-Akt ↓, ROS ↑	[139,140,141]
K562 human chronic myelogenous leukemia cells; HepG2 human hepatocellular carcinoma cells; HCT 116 human colorectal carcinoma cells; Sarcoma 180 murine sarcoma cells; B16F10 musculus skin melanoma cells; DLA Dalton’s lymphoma ascites cells; Swiss albino mice—Dalton’s lymphoma cells;	In vitro bromelain + peroxidase treatment In vivo (oral administration) bromelain + peroxidase treatment	PCD induction: p53 ↑, Bad ↑, Bax ↑, Bcl-2 ↓, ROS ↑, Caspase-3 ↑, cytochrome c ↑, NF-κB ↓	[148,149,150]
MKN45, KATO-III gastrointestinal carcinoma cells; HT29-5F12, HT29-5M21, LS174T colon adenocarcinoma cells;	In vitro bromelain or bromelain + N-acetylcysteine treatment	PCD induction: Caspase-3 ↑, Caspase-7 ↑, Caspase-8 ↑, Caspase-9 ↑, cytochrome c ↑, cleaved PARP ↑, Bcl-2 ↓, p-Akt ↓, MUC1 ↓, p53 ↑ Cell cycle arrest: cyclins A, B, D and E ↓ Autophagy induction: LC3BII ↑, beclin 1 ↑	[142,143]
FK-1, SZ-1 cholangiocarcinoma (CC) cells	In vitro bromelain treatment	Decrease in the proliferation, invasion, and migration of CC cells PCD induction: cleaved PARP ↑, p-Akt ↓, NF-κB ↓, alterations in the expression of MAPK family proteins: ERK ½ ↓, STAT-3 ↓, Changes in epithelial-mesenchymal transformation: E-cadherin ↑, N-cadherin ↓	[144]
DLD-1, HT-29, HCT116 human colorectal cancer cells	In vitro bromelain treatment	PCD induction: Caspase-3 ↑, Caspase-8 ↑, Caspase-9 ↑, apoptosis inducing factor (AIF) ↑, endonuclease G (Endo G) ↑, cleaved PARP ↑, ROS ↑, Autophagy induction: LC3BI/II ↑, beclin 1 ↑, p62 ↑, ATG5/12 ↑,	[145]
Caco2, CT116, G13D human colorectal cancer cells; KRASG12D mutant heterozygous mice	In vitro and in vivo bromelain treatment	Ferroptosis induction: accumulation of lipid-based ROS, Long-chain-fatty-acid—CoA ligase 4 (ACSL4) ↑	[146]
DLD-1, Caco2 human colorectal cancer cells; Male imprinting control region mice	In vitro and in vivo bromelain treatment	PCD induction: p-Akt ↓, Caspase-3 ↑, Caspase-7 ↑, ROS ↑, alterations in the expression of MAPK family proteins: ERK ½ ↓, Reduction in the development of aberrant crypt foci, polyps	[147]
FPAC, ASPC1, HEP3B, HEPG2 human pancreatic cells; AGS human gastric carcinoma cells; PC3 human prostate carcinoma cells; MCF7 human breast Adenocarcinoma cells; Mice challenged with 4T1 triple-negative breast cancer cells; MDA-MB231 human breast adenocarcinoma triple-negative breast cells; PET, YOU malignant peritoneal mesothelioma cells;	In vitro bromelain/bromelain + N-acetylcysteine with combination with Gemcitabine/5-fluorouracil/Oxaliplatin/Doxorubicin treatment In vivo bromelain + cisplatine treatment In vivo bromelain + cisplatine treatment In vitro bromelain + cisplatine/5-FU	The synergistic action of the mixture: cell proliferative and colony formation inhibition, reduction the doses of chemotherapeutic agents The synergistic action of the mixture: modulation the tumor environmental Inflammation—Gremlin ↓, IL-1β ↓, IL-4 ↓, NF-κB ↓ Bcl-2 ↓, Bax ↑ The synergistic action of the mixture: Caspase-3 ↑, Caspase-7 ↑, Caspase-8 ↑, Caspase-9 ↑, cytochrome c ↑, Bcl-2 ↓, cleaved PARP ↑, p-Akt ↓, MUC1 ↓, LC3BI/II ↑, NF-κB ↓,	[155,163,164,165,166,167]
HT1080, SW872, VA-ES-BJ, SW982 human sarcoma cells;	In vitro bromelain + N-acetylcysteine treatment	PCD induction: Caspase-3 ↑, Caspase-8 ↑, cleaved PARP ↑, Bcl-2 ↓, Bax ↑ Autophagy induction: LC3BI/II ↑	[156]
SCC25 human oral squamous carcinoma cells; Ca9-22 human oral squamous carcinoma cells	In vitro bromelain treatment	PCD induction: Increase in the population of Sub-G1 cells, p53 ↑, Caspase-3 ↑, Caspase-7 ↑, Caspase-9 ↑ cleaved PARP ↑, Bcl-2 ↓, Bax ↑, Lamin A/C ↓, cytochrome c ↑, AIF ↑	[157]

The effects of bromelain are marked as follows: ↓ decreased, ↑ increased.

## Data Availability

Data sharing not applicable.
